# Ransomware detection using deep learning based unsupervised feature extraction and a cost sensitive Pareto Ensemble classifier

**DOI:** 10.1038/s41598-022-19443-7

**Published:** 2022-09-19

**Authors:** Umme Zahoora, Asifullah Khan, Muttukrishnan Rajarajan, Saddam Hussain Khan, Muhammad Asam, Tauseef Jamal

**Affiliations:** 1 Department of Computer and Information Sciences, Pakistan Institute of Engineering and Applied Sciences, Nilore, 45650 Islamabad Pakistan; 2 PIEAS Artificial Intelligence Center (PAIC), Pakistan Institute of Engineering & Applied Sciences, Nilore, 45650 Islamabad Pakistan; 3 Center for Mathematical Sciences, Pakistan Institute of Engineering and Applied Sciences, Nilore, 45650 Islamabad Pakistan; 4 School of Mathematics, Computer Science and Engineering, City University of London, London, EC1V 0HB UK; 5 Department of Computer Systems Engineering, University of Engineering and Applied Sciences, Swat, 19060 Pakistan

**Keywords:** Biological models, Genetic techniques, Evolution, Computer science

## Abstract

Ransomware attacks pose a serious threat to Internet resources due to their far-reaching effects. It’s Zero-day variants are even more hazardous, as less is known about them. In this regard, when used for ransomware attack detection, conventional machine learning approaches may become data-dependent, insensitive to error cost, and thus may not tackle zero-day ransomware attacks. Zero-day ransomware have normally unseen underlying data distribution. This paper presents a Cost-Sensitive Pareto Ensemble strategy, CSPE-R to detect novel Ransomware attacks. Initially, the proposed framework exploits the unsupervised deep *Contractive Auto Encoder (CAE)* to transform the underlying varying feature space to a more uniform and core semantic feature space. To learn the robust features, the proposed CSPE-R ensemble technique explores different semantic spaces at various levels of detail. Heterogeneous base estimators are then trained over these extracted subspaces to find the core relevance between the various families of the ransomware attacks. Then, a novel Pareto Ensemble-based estimator selection strategy is implemented to achieve a cost-sensitive compromise between false positives and false negatives. Finally, the decision of selected estimators are aggregated to improve the detection against unknown ransomware attacks. The experimental results show that the proposed CSPE-R framework performs well against zero-day ransomware attacks.

## Introduction

Ransomware is a specific extortion attack that exploits cryptography to hijack a victim’s computer and consequently mandate ransom payment for disinfecting the infected resource^1^. Nowadays, due to monetary benefits, these kinds of extorting attacks have been emerging very rapidly and causing high financial losses to individuals and organizations. The first ransomware attack, known as *Acquired Immunodeficiency Syndrome (AIDS)* or Trojan-PC Cyborg, was reported in 1989. It spread through twenty thousand septic floppy disk drives dispersed to the AIDS conference accomplices^2^. It stays silently in the system and triggers after 90 times the system’s reboot. Upon activation, it either encrypts the files or hides the directories. At that time, due to less connectivity, it did not proliferate on a large scale. Later, ransomware intruded cyber resources utilizing different forged applications. These fraudulent applications grab users by falsely notifying them that the user’s critical data is breached and demand ransom for data recovery. In the year 2015, the *Federal Bureau of Investigation (FBI)* reported an 18 million US dollars loss due to ransomware attacks^3,4^. One of the latest cyber-attacks in May 2017, conceded via WannaCry malware, shows that ransomware has established over the years and does not use the conventional propagation methods anymore. According to one of Kaspersky's reports, 62 novel ransomware families were detected at the beginning of year 2016^5^. In 2016, an 11% increase in ransomware attacks was observed as compared 2015. Organizations and individuals practice different types of detection systems against these attacks. However, the financial benefits are motivating attackers to escalate the production of new variants to evade the security of the existing systems. Although zero-day vulnerabilities are exploited in a variety of attacks, but when combined with ransomware, it can be more devastating. Zero-day attacks are increasing very rapidly with an increase in the applications. According to cybersecurity ventures, increase in zero-day attacks observed in 2015 is one attack per week, which may increase to one attack per day in 2023^3^. Subsequently, it could lead to monetary loss, reputational impairment, and regulatory charges.

Numerous studies have presented solutions for ransomware detection. Normally, antivirus systems are signature-based that compares the inbound file to a prearranged list of malware signatures^4,6^. However, these signature-based methods are not effective against zero-day malware detection. To respond to the shortcomings of signature based detection, various static and dynamic analysis based detection system are presented in literature. Static analysis based detection systems examine an inbound file without execution. These methods are relative fast but are unable to detect the polymorphic attack modalities. In contrast, dynamic analysis based methods examine an inbound file in a virtual environment during its execution^7^. However, this is a very time-consuming process to execute each inbound file in a virtual environment. Another approach is to analyze only specific ad-hoc events^8^ to save the time. Particular events based procedures are not suitable as they might not trigger or may happen when the process is lasting. Thus, such methods undergo a high false alarm rate and low detection rate. Anomaly-based methods are one class-based machine learning method that try to learn “normal” behaviour, and then any deviation is flagged as malicious activity^9^. Anomaly detection is susceptible to a high false-positive rate. In particular, conventional approaches considers the misclassification errors to be of equal cost^9,10^. However, in various real-world solicitations, this assertion may not be the case. The cost differences between the false positive and false negative may vary and can be relatively large. For example, in intrusion detection, all intrusions are not equally vulnerable. In most vulnerable malware cases, false alarm cost is less costly than that of false negative rate and thus requires developing an *Intrusion Detection System (IDS)* that is more sensitive to detect vulnerable malware. One such example of the most vulnerable malware is ransomware.

A current research trend is generally to use *Deep Learning (DL)* capabilities to generalize models and automatic feature extraction from raw data^11^. Due to these distinctive features DL has proven its effectiveness in many fields such as image recognition, recommendation systems, emotion analysis^12^ and speech recognition. Existing DL based detection systems relies on the assumption that underlying data distribution of unseen class of attack is similar to the seen class of attack. Very few DL researches in the literature aims to detect zero-day attacks^13–16^. DL based methods are data-dependent techniques. Therefore, some recent studies have addressed the problem of data scarcity by proposing methods based on few-shot learning. Similarly, Zhu et al.^17^ exploit meta-learning and Neural Network to present a few-shot ransomware detection method. Wang et al.^18^ proposed a multi-porotype based few shot learning technique that models each family of malware separately, in order to increase the generalization capacity. These works are quite interesting and have achieved improved performance, and have validated the practicality of the concept of few shot learning in intrusion detection, however, there are still challenges of implementing these methods in the case of zero-shot samples.

In this paper, we propose to explore the capabilities of *Deep Contractive Autoencoder (DCAE)* to serve as core feature extractor. The main goal is then to build an ensemble based ransomware detection system that can detect new ransomware attacks, with a high recall and low false-positive rate. Therefore, having a high detection ability of zero-day attacks can thus reduce concerns related with novel attacks. The contributions of this study are as such:

1. This work presents a multi-phase novel framework named “CSPE-R” against *zero-day Ransomware detection.*
2. First, the proposed framework is able to explore low-dimensional core embeddings through the proposed *Core Feature Hunting (CFH)* module. Therefore, the contractual penalty in the loss function is improved to suppress small variations. This minimizes the effect of variations in different ransomware families and thus, enables it to separately learn the basic semantic features of ransomware as well as goodware classes.
3. The proposed framework is able to extract multiple core feature vectors by changing the bottleneck layer of the DCAE and thus, the dimensionality of the core feature vector. This in turn helps in capturing multiple characteristics of a single class of attacks.
4. Multiple cost-sensitive predictors are then trained for each core feature vector to learn these various characteristics of the ransomware variants and to solve the class imbalance problem.
5. Finally, a new Pareto-optimality based selection strategy of the base estimators is developed to achieve a cost sensitive compromise among the false positives and false negatives. The selected classifiers are then aggregated to make a ransomware sensitive detection system.

### Related work

Enduring commercial mitigation strategies are based on classic signature techniques^19^—for example, SurfRight’s HitmanPro.Kickstart employs real-time forensic analysis to match the potential file with known ransomware strains. Similarly, Avast’s Ransomware Removal tool is dependent on the naïve ransomware families to access the key for releasing encrypted files. However, these keys are not generated based on each infection^20^. Other, most popular commercial IDS employs pattern matching based detection, e.g. YARA, Suricata, and Snort. These Signature-based and pattern matching methods lack generality; therefore, they can only detect the subset of malware and ineffective to detect zero-day attacks. However, generally static analysis methods like packers and *Portable Executable *(*PE*) analysis tools can extract useful information about malicious binary.

Schultz^21^ is a known pioneer who pursued malware detection on particular static features such as PE header attributes, byte arrangements and mined strings by employing data mining methods. He compared the data mining methods with Signature-based classical approaches and achieved a detection rate of 97.76% on test data that is much better than the Signature-based methods. Similarly, Shabtai et al.^22^ extracted static features from the PE file header to develop a malicious files detection system. He further categorized the extracted features into six broad categories: version number, PE header, import, optional header, export, and resource. They also concluded that a single classifier trained on a single feature is ineffective and suggests that training a weighted average ensemble on different features can give better results. Young^23^ used five particular fields: Section Name, Checksum, Set data dimensions, *Dynamic Link Library (DLL)* features, major image version. However, these static analysis based intrusion detection methods are ineffective for analyzing the specific behaviour of ransomware. Zhang et al.^24^ offered a static analysis-based SA-CNN Crypto-ransomwares detection system. Arp et al.^25^ presented DREBIN to detect malicious code on Andriod mobile. The authors extracted static features, e.g. Ip addresses, sensitive *Application Programming Interface (API)* calls and permissions from application *APK’s (Android Package Kit)* and trained Linear *Support Vector Machine (SVM)* to discriminate the malware and benign samples. However, it could not detect the run time loaded applications.

Though ransomware threats are proliferating, and the Windows operating system has been their supreme prevalent goal. There are relatively very few researchers focusing on Windows ransomwares. Das et al.^26^ presented a frequency centric model that groups the similar API and then calculates their frequencies to distinguish the benign and malicious samples. RAMD presented by Asghar et al., is an anomaly-based malware detection method that model the registry-based behaviour of benign samples. Therefore it looks for abnormal registry accesses. In detail, RAMD^27^ is an ensemble-based method that trains multiple one class base classifiers at the initial stage. In Next stage uses a swarm intelligence pruning algorithm to select only those classifiers that have diverse output and high detection accuracy. Further, to combine the outputs of selective ensemble classifiers, RAMD uses *Fibonacci-based super increasing ordered weighted averaging (FSOWA)* as aggregating operator. On the other hand, RAD, proposed by Apap et al.^28^, is an incongruity exposure method that screens the abnormal registry entrees utilizing five low-level structures. These Low-level features are selected from each registry access. They build a detection model trained on these low-level behavioural registry features using the probabilistic Anomaly algorithm. However, when a process is at a running state, it may have too much registry access. Therefore it is a time-consuming process to select low-level features from each registry access.

In literature, some of the researchers exploited deep learning models to develop effective detection systems. Deep Neural Networks architectures are generally established on the supposition that by increasing depth, the representation capability can be increased. These networks tend to have more non-linear mappings, and thereby enhancing the features hierarchies and thus providing more abstraction of the problem to be learned. Tian et al.^29^ proposed a deep learning based ensemble technique for web attack detection. Ding et al.^30^, designed Deep Belief Network (DBN) work for malware detection and used Opcode sequence as an important feature. On the other hand, utilizing RNN-Auto Encoders (AE), Xin Wang generated File Access sequence by tracing API call sequences and evaluated its role in the classification of malware. Similarly, Tian et al*.*^31^ exploited deep learning in distributed environment for edge devices that is responsible for concurrent learning and improvement of the edge devices. The role of deep learning and ensemble learning is evident in the field of intrusion detection, however efforts are still needed at efficiently classifying zero-day ransomware variants.

## Materials and methods

**Figure 1:**
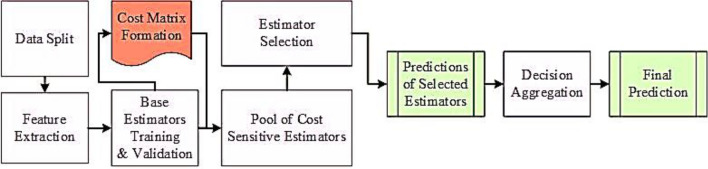
Abstract diagram of the proposed CSPE-R system.

This work suggests a multi-phase novel *Cost-Sensitive Pareto Ensemble framework* named “CSPE-R” against *zero-day Ransomware detection*. The workflow is decomposed into 5 phases: (1) core features hunting, (2) cost matrix formulation, (3) learning heterogeneous base estimators, (4) estimator selection, and (5) decision aggregation. The general context of the suggested model is displayed in Fig. 1.

### Novelty, motivations, and advantages of the proposed method

This section discusses the rationale of using our design choices to detect zero-day ransomware and handle its related challenges. In the proposed approach, we exploit the heterogeneous runtime events as an alternative to static and specific events, as in the static analysis features may not always detect the attack modalities. Also, particular events are not suitable as they might not happen, or happen when the process is terminating.

Although Deep Learning has been extensively studied in other fields^32–34^ and obtained significant results, Contractive Autoencoder is still in its infancy of development in the field of intrusion detection and only limited studies have been conducted focusing on the ransomware classification. The proposed method seek to extract multiple core features for zero-day attack detection. As, by applying multiple core features, heterogeneous properties of the attack can be utilized and by learning a classifier on each core features set, and training them using heterogeneous ensemble, the overall performance can be enhanced. The proposed multi-core feature based Deep Contractive Autoencoder not only outperforms single-core features based on deep Contractive autoencoder but also has superior efficiency and generalization performance.

Another big challenge in identifying unknown ransomware attacks is data scarcity. An appropriate choice to handle these challenges is to utilize the ensemble learning method, as it is normally more generalized and robust compared to a single classifier. Ensemble learning methods retain numerous valuable features and are considered to be robust to noise, scalable, and incremental. In case of data scarcity, it is challenging to hybridize the ensemble of deep learning models; therefore, the proposed CSPE-R method exploited both structural and empirical minimization benefits (by using SVM, *Random Forest* (RF), and *Logistic Regression Classifier* (LRC) as base classifier) to increase the zero-day ransomware detection performance. Additionally, to learn the ransomware specific patterns, a new cost sensitive ensemble based novel estimator selection strategy is developed. This novel estimator selection method systematically incorporates multi-objective non-dominant sort to select only high recall estimators. Finally, the decisions of the selected estimators are grouped.

### Dataset

**Figure 2:**
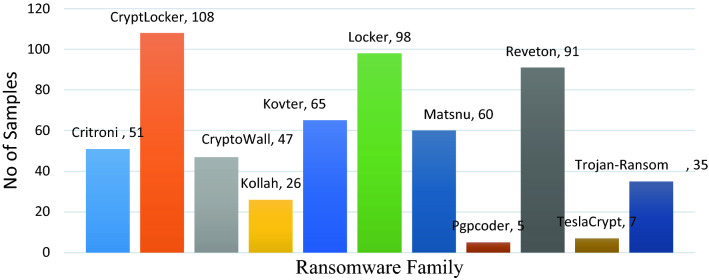
Ransomware family vs. no of samples.

We used the dataset developed by Sgandurra et al.^35^ established in February 2016 as a training dataset to construct the EldeRan prediction model. The dataset is obtained by analyzing the samples in a sandboxed environment. The dataset comprehensively includes the dynamic behaviour of running the sample in a safe environment. In the dataset, malicious samples are labelled as ransomware (one or positive class), and benign samples are labelled as goodware (zero or negative class). It contains of 582 ransomware and 942 goodware instances. Ransomware samples are further classified into 11 families. Figures 2 show the details of ransomware families used in the dataset. The assembled samples are the most widespread modifications of the ransomware, and the mainstream is CryptoRansomware. The goodware samples are prepared from reliable sources. Goodware samples comprise file utilities, browsers, emulators, drivers, word office gears, games applications, etc. Separately these applications are executed for thirty seconds in a sandbox setting. Sgandurra et al. considered only host based features while structuring the samples. The used dataset includes the following categories of features: (1) Windows API calls, (2) Registry Key related operations, (3) file opening, reading and deletion, (4) alignment of system file operation according to their extensions. (5) directory creation and enumeration operations, (6) files dropped by a particular application, and (7) binary strings. Unlike the methodologies discussed in the literature, the CSPE-R methods' focus is not on a single event. It is established using a combination of dynamic features like files extentions, API requests, registry keys set-ups files extension, file directories set-ups, drop files record, strings, and file processes.

We split the ransomware families into seen and unseen classes to build a robust zero-day ransomware detection model as described in Table 1. Where the seen, families are used for training and validation purposes, and the unseen classes are reserved as zero-day attacks for testing purposes. Finally, we obtained 1257 samples in the training dataset, including 448 ransomware and 809 goodware, to build the proposed prediction model.

**Table 1:** Train test split of ransomware families.

Seen classes	Unseen classes
Citroni	Pgpcoder
CryptLocker	Reveton
Cryptowall	TeslaCrypt
Kollah	Trojan Ransom
Kovter	
Locker	
Matsnu	

### Contractive autoencoder

The objective of the autoencoder based Deep Neural Network is to learn the complex features representations in more abstract form. Auto-encoder as its feature extraction application^26–30^, can be define as a function with particular parameters in closed form. Let this function be denoted as $g\theta$ which is the encoder function that will compute the feature vector $v = g\theta \left( i \right)$ from input feature vector $i$. For every instance $i\left( k \right)$ from a data set $\left\{ {i\left( 1 \right),...,i\left( K \right)} \right\},$ we define

1 $$g\theta \left( i \right) \, = \, \partial \left( {b \, + \, W_{i} } \right)$$

where $g\left( k \right)$ is representing the learned representation or transformation from $i\left( k \right).$ Autoencoder uses another decoder function to map this transformation back to original input defined as $z\theta$.This function produces reconstruction $d \, = \, z\theta \left( v \right).$

2 $$z\theta \left( v \right) \, = \, \varphi \left( {d \, + \, W_{v} } \right)$$

In general, learning process involves the learning of the set of parameter $\theta$ simultaneously for reconstructing the input space with the objective to incur the lowest reconstruction error $O\left( {i,d} \right)$ using stochastic gradient. However, these generated abstract features due to compression in reconstruction phase may lost some useful information. To this end, the goal of CAE is to learn the robust representation, which is less sensitive to small changes in data. That is achieved by using an additional regularization term $$\lambda \left\| {J_{h} (k)} \right\|_{\begin{subarray}{l} F \\ \end{subarray} }^{2}$$ that penalizes the input for little changes, this term is also known as sensitivity penalization term. $J\left(k\right)$ is the Jacobian matrix of encoding layer (*h*) with respect to input function *(x).* It is calculated by finding the partial derivatives of *h w.r.t* x. Further by summing up the squares of the derivatives the generated mapping persuasively contracts the data. ${^{\prime}}\lambda{^{\prime}}$ is the penalty parameter that controls the contraction of the Jacobian term. Hence it penalizes the large derivative that ménages the degree of variation in input concerning the learned hidden representation. This regularization term provides the approximation of local density function that is assistive in selecting a single vector which is a tangent plane to the concentrated region of manifold. Furthermore, it’s under complete version is used to capture the salient structure of the input data distribution.

3 $$JAE\left( \theta \right) \, = \, I \, k \, O\left( {i\left( k \right),z\theta \left( {g\theta \left( {i\left( k \right)} \right)} \right)} \right) + \lambda \left\| {J_{h} (k)} \right\|_{\begin{subarray}{l} F \\ \end{subarray} }^{2}$$

### Proposed deep features hunting technique

In the feature extraction phase, we have proposed a *Core Feature Hunting (CFH)* approach that utilizes Undercomplete deep *Contractive Autoencoder (CAE)* to extract the core ransomware features. CAE, is an unsupervised generative model that is trained to learn a single uniform distribution of various ransomware attacks. It aims to reconstruct the original data distribution by retaining some valuable variations. The proposed CFH module is trained and validated in an unsupervised manner to minimize the reconstruction loss on core features. The optimized architecture constitutes ten hidden layers, including five encoding and decoding layers. The first five encoding layers encode the original features to core semantic features. While the last five decoding layers, decode the encoded features to reconstruct the original input by learning an optimized loss function described in Eq. (3). In encoding layers, each consequent layer constituents 16,382, 4000, 2000, 1200, 600, and 100 neurons. In contrast, the decoding layer constituents 600, 1200, 2000, 4000, and 16,382 neurons in each layer separately. The penalty term of the loss function is optimized to get smaller derivate to suppress the family variations of ransomware.

**Figure 3:**
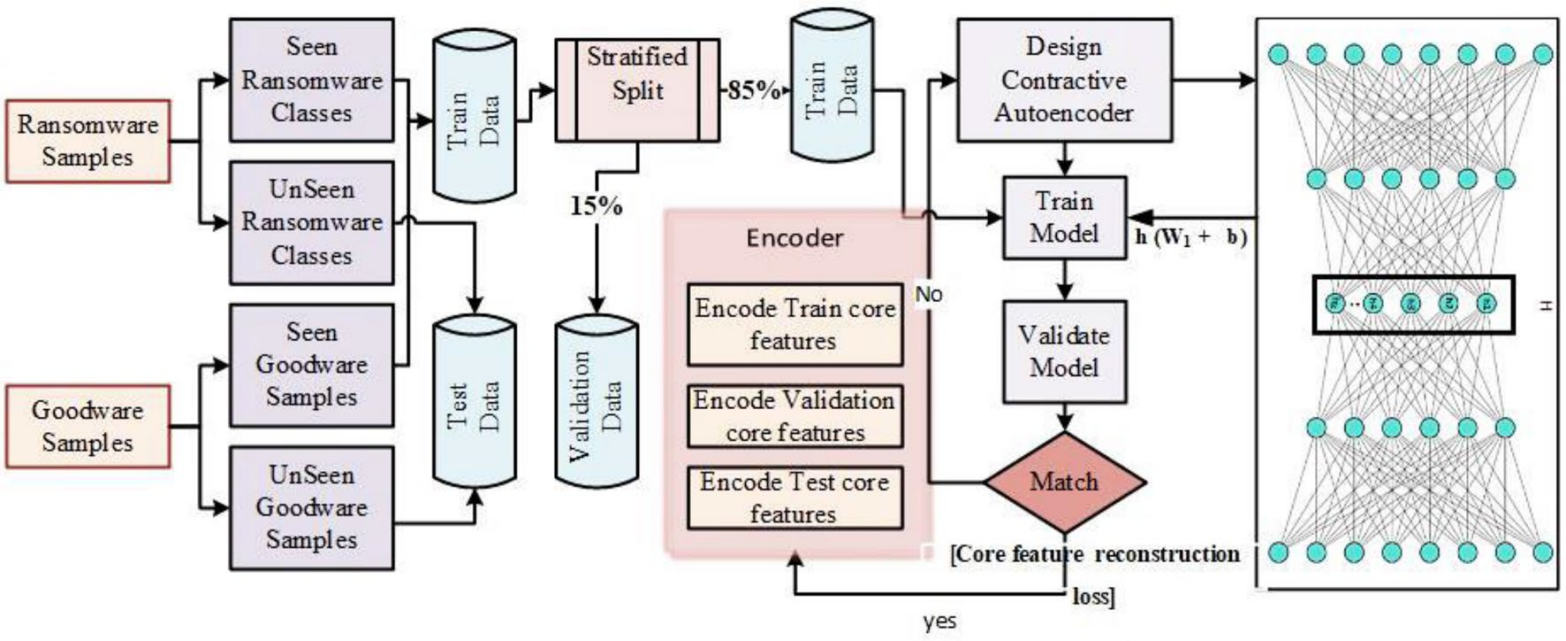
Core feature hunting using CAE.

The proposed method models the uniform encoding representation of data in two feature spaces by varying the number of neurons in the designed topology. Therefore, we trained two CAE models to obtain two reduced representations. One representation contains 100 neurons in the bottleneck layer (last encoding layer), while the other representation contains 500 neurons in the bottleneck layer. Due to different dimensions of each core feature vector, they possess different level of details. Figure 3 show the detailed procedure of hunting the ransomware core features. Initially, we divided the ransomware and good ware samples into train, test, and validation data sets as described in Sect. 3.2. Then, trained and validated the CAE with training data and validation data, respectively. CFH was trained using SGD as an optimizer. CAE models were trained for 100 epochs with a weight decay of 1e−4 and preliminary learning rate of 1e−2. The batch size, and lambda value were set to 5, and 0.000001, respectively. CAE models were optimized by minimizing the contractive loss function. Finally, the model having the minimum reconstruction loss for validation data, was used to encode the original data.

### Ransomware sensitive heterogeneous base estimators

In case of data scarcity, it is challenging to hybridize the ensemble of deep learning models. Therefore, the proposed CSPE-R method exploited both structural and empirical minimization benefits by training multiple classifiers such as SVM, *Random Forest (RF),* and *Logistic Regression Classifier (LRC)* on derived deep features. As, classifiers with similar training performances may have different generalization results^38^. The set of classifiers, when worked together, can explore a more exhaustive solution space. Therefore the proposed technique ensemble the heterogeneous base estimators, e.g. cost sensitive SVM, weighted LR, and cost sensitive RF, to overcome the cost indifference and class imbalance problem. These ransomware sensitive base estimators are trained on the derived semantic core features of sizes 100 and 500. The parameter configuration of these classifiers is carried out using five cross-validations. Parameter values obtained after optimization are: SVM (C = 100, kernel = , gamma = 0.1.), RF (max_features = 'auto', n_estimators = 700, max_depth = 9, criterion = 'gini') and LR(C = 0.1, penalty = 'l1', tol = 0.01). These models are optimized on precision and recall due to highly imbalance data. To resolve the issue of imbalance and high priority minority class derived the cost matric for different class. Table﻿ 2 shows the derived cost matrix for different classifiers.

**Figure 4:**
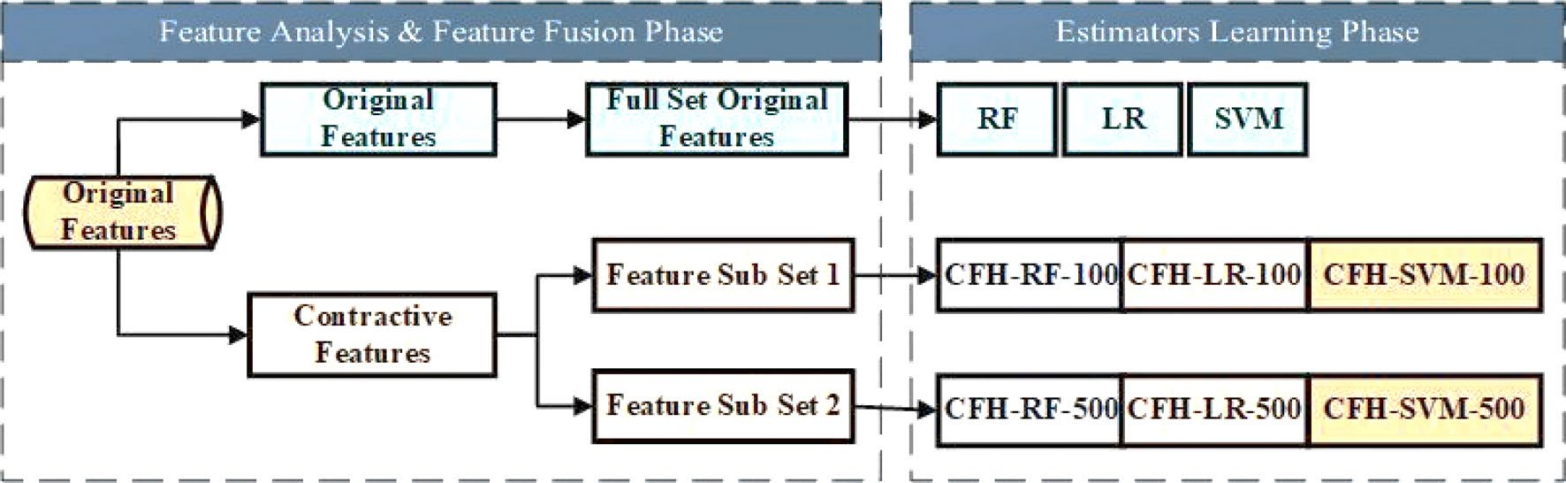
Heterogeneous estimators learning on different feature spaces.

The detailed proposed ensemble strategy is described in Fig. 4. Also, the proposed CSPE-R system ensemble the conventional base estimators that are insensitive to the type of error cost, e.g. RF, LR, and SVM, are trained on original features.

### Cost matrix formation

The optimal cost matrix of different base estimators shown in Table 2 is made by using the five cross-validation method. The ransomware dataset was separated into seen classes and unseen classes, where the training classes (seen classes) and test classes (unseen classes) are disjoint to ensure zero-day attack in the test phase. All the base estimators were trained with the seen class dataset, class weights, and additional hyperparameters alignments were designated based on estimator average performance on the five-fold validation dataset.

**Table 2:** Cost matrix of errors.

Models	FN	FP
SVM	2	8
RF	6	12
LR	2	16

### Creating a ransomware-sensitive CSPE-R ensemble

**Figure 5:**
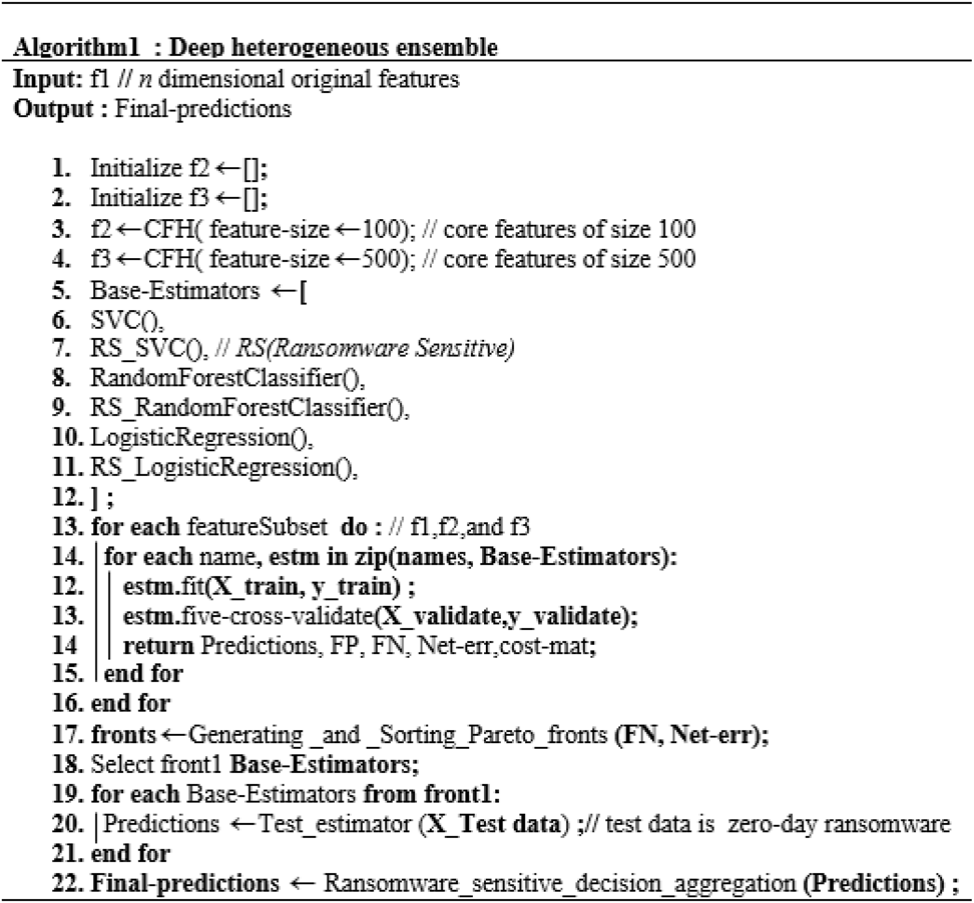
Algorithm 1: Creating deep heterogeneous ensemble.

The proposed technique CSPE-R aims to effectively deal against zero-day malware detection challenges, e.g. malware modalities, scarcity of data and class imbalance. The proposed CFH methods exploit CAE's uniform encoding abilities and refine the generalization of the detection system through heterogeneous ensemble training. The proposed ensemble is established on five steps. In the first step, CFH is proposed to extract core features that are invariant to the slight variations present in emerging ransomware variants. In the second and third steps, base-learners are trained, and the cost matrix is formed using different feature spaces that deal with data scarcity and class imbalance problems. Each feature space is trained using three different classifiers. Each sub-feature space can be generated and trained independently. Therefore, this step can be performed in parallel to save time. The return of the second step is the set of predictions that are further sorted in step 3 using non-domination sort to select the optimal base learners. For accurate base-learners, each base learner is trained using five cross-validations on precision and recall. In step 4, the selected base learner's decision is combined using the ransomware-sensitive combination rule. The detail of the CSPE-R steps is provided in the form of pseudocode in Algorithm 1 Fig. 5.

### Proposed Pareto optimality based estimator selection strategy

The ensemble selection process is inherently a multi-objective problem that possess two or more objectives simultaneously e.g. maximizing the classifiers diversity, maximizing the classifier’s accuracy, minimizing error and minimizing the number of base classifiers so on^39^. All these goals are conflicting, as insufficient number of base estimators lead to decline in accuracy and gain in error. In order to achieve these objectives simultaneously, previous ensemble selection approaches can only deal with a single cost function. But more recently, it has been revealed that, explicit formulation of each goal is more effective this is due to the effective application of multi-objective genetic algorithms in the field of machine learning such as features optimization,pareto-optimization, clustering etc. The proposed ensemble selection technique simultaneously optimizes false negative and net-error. As a substitute of optimizing a single cost function of these two objectives, the proposed method consider explicitly optimizing the bi-objective ensemble selection problem, which is expressed as:

4 $$arg \, mins \, \left( {fN\left( H \right),{\text{ N}}et - error\left( H \right)} \right)$$

where, *H* is the selected base estimator. The output of this function is not a scalar value but an objective vector that is assigned to each candidate solution. The vector comparison among two candidate’s solutions not as simple as in the case of scalar value, since it is may be the case that one candidate solution is superior on the first objective while the other is superior on the second objective. Therefore, solution to muti-task problem is a number of optimal solution instead of single optimal solution. This motivated us to use this Pareto-optimality to find multiple optimal base learners instead of a single base learner. Therefore, to compare two objective vector the domination relationship is usually considered.

### Domination relationship in case of bi-objective minimization:

**Figure 6:**
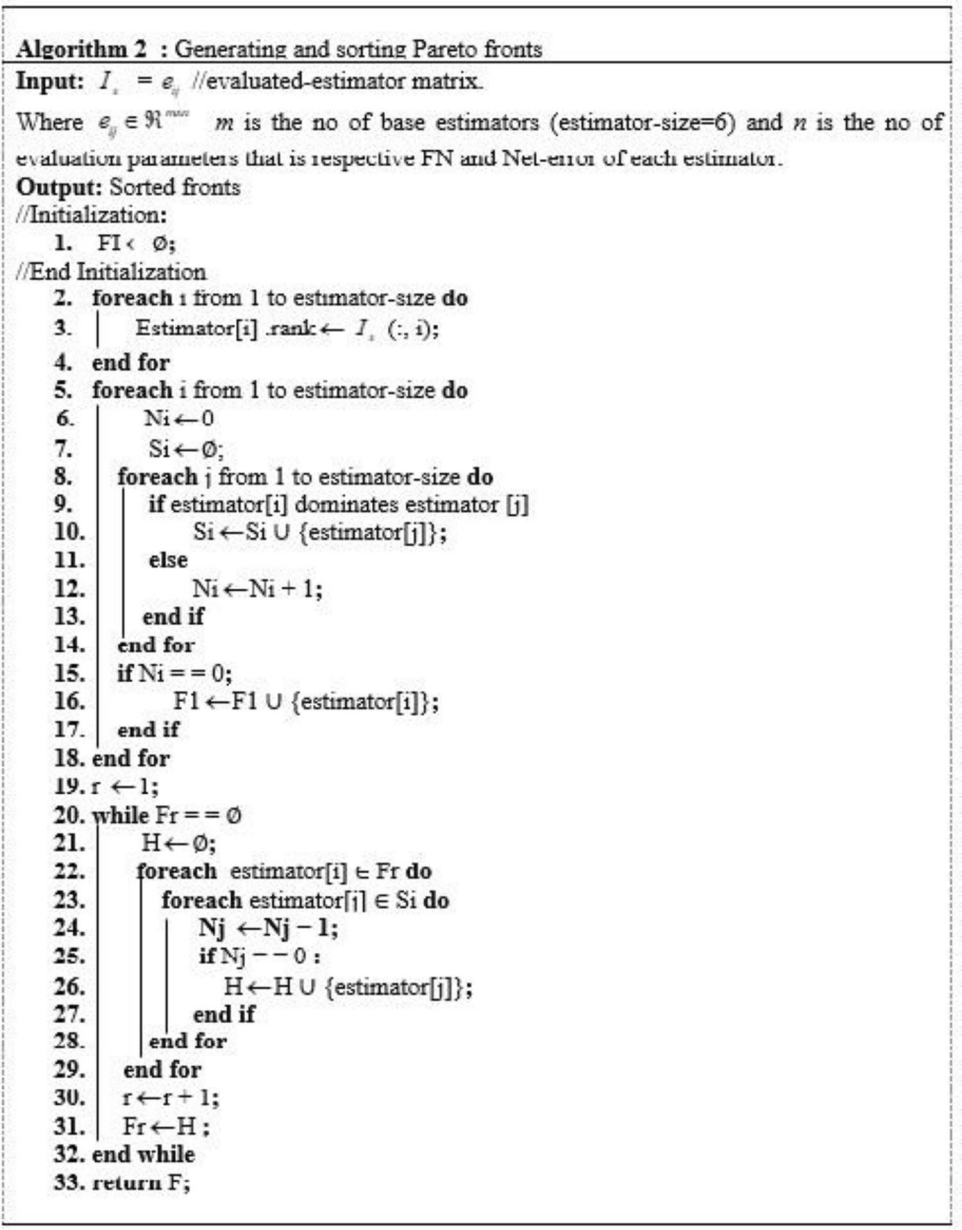
Algorithm 2: generating and sorting Pareto-front.

**Figure 7:**
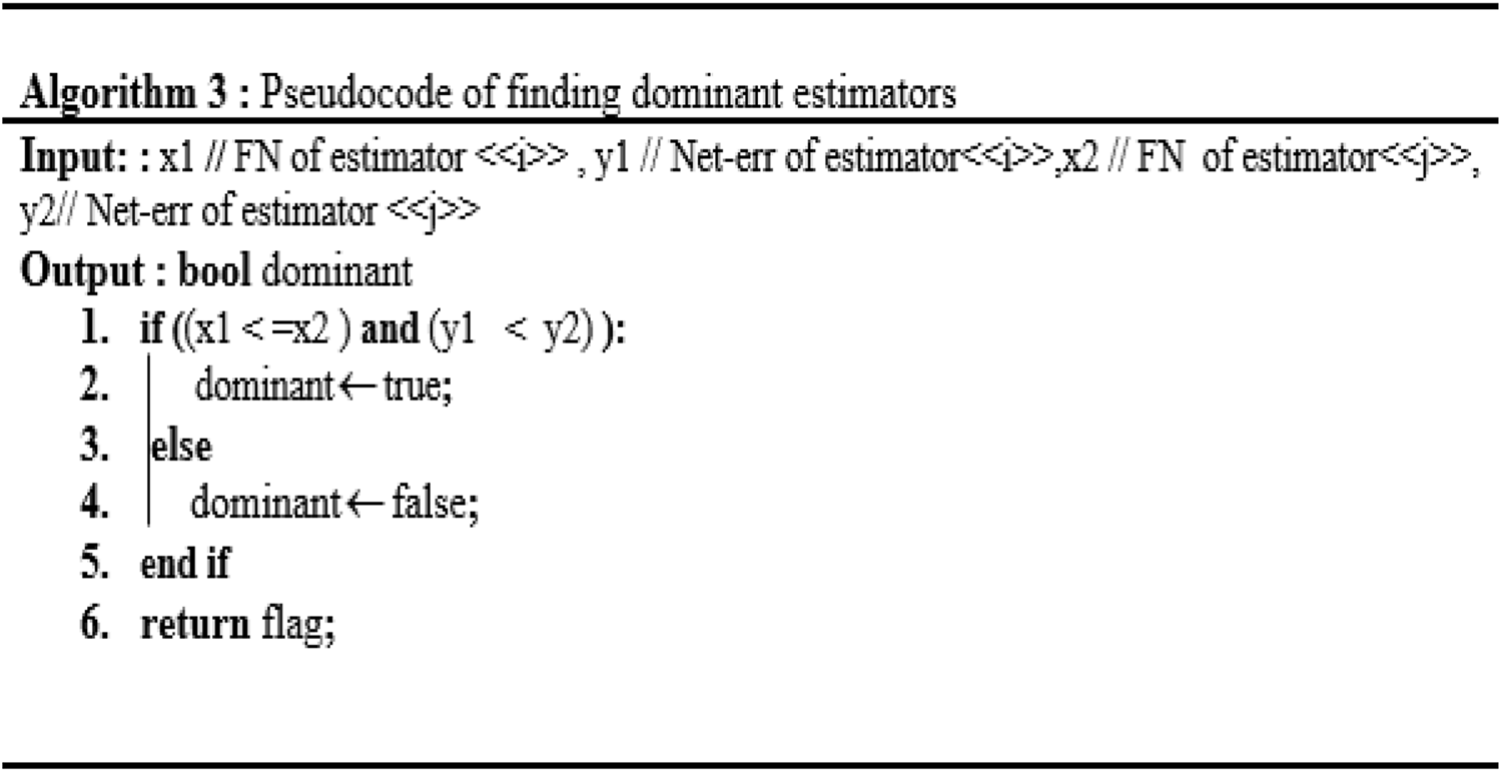
Algorithm 3: Pseudocode of finding dominant estimators.

Let $O = \{ o_{1} ,o_{2} \} :C \to \Re^{2}$ be the objective vector meant for two candidate solutions $c_{1} ,c_{2} \in C$ then:

1. $c_{1}$ weakly dominates $c_{2}$ if $o_{1} (c_{1} ) \le o_{1} (c_{2} )$ and $o_{2} (c_{1} ) \le o_{2} (c_{2} )$ denoted as $s\underline{ \succ } o$.
2. $c_{1}$ dominates $c_{2}$ if $s\underline{ \succ } o\quad$ either $o_{1} \left( {c_{1} } \right) < {\text{o}}_{1} \left( {{\text{c}}_{2} } \right) \, or{\text{ o}}_{2} \left( {c_{1} } \right) \, < o_{2} \left( {{\text{c}}_{2} } \right),$ denoted as $s \succ o$. A solution c is Pareto optimal if there is no other candidate solution in C that dominates c. These set of solutions are divided in various fronts based on the dominance comparison. Where all candidate solutions of front 1 dominates the solution of all other subsequent fronts. This work, propose the Pareto optimality based ensemble selection procedure (as described in Figs. 5, 6 and 7) to resolve a considerable compromise between FP and FN. Pareto-optimality based multi-objective learning enable user to build optimal learning models without considering the hyperparameters values before training. Pareto-optimality is motivated by an evolutionary multi-genetic algorithm, which has been recognized for approximating some NP-hard problems^36,37^. Once a Pareto optimal solutions set to the bi-objective origination of ensemble selection has been resolved, we can choice out one absolute solution agreeing to our preference, which is done by combining the classifiers of front1 solution.

### Decision aggregation

A decision aggregation scheme combines the output of multiple base estimators into a single result. The proposed decision scheme bifurcates between ransomware and goodware. This methodology aggregates the decision of selected heterogeneous estimators by OR logic. It is flagged as ransomware positive if any of the estimators mark it. Otherwise, it is goodware.

### Implementation details

The experimentations of the proposed method were carried out on desktop machine having an Intel® Core™ i7-33770 processor, 16 GB RAM, 64-bit window 7 Professional operating system and used python 3.7 to implement the various modules of the proposed techniques. However, the base estimators were developed using Scikit library. CFH module was developed in Keras library. Moreover, excel, matplotlib, and pillow libraries were used for plotting graphs.

#### Performance assessment measures

The developed framework is evaluated using following evaluation matrices.

4 $$\, Precision \, \; = \; \, TP/TP\; \, + \; \, FP$$

5 $$Recall \, = \, TP/TP \, + \, FN$$

6 $$Accuracy = TP \, + \, TN/ \, TP \, + \, TN \, + \, FP \, + FN$$

where, *True Positive (TP)* is predicted positive the real positive sample, *False Positive (FP)* is predicted positive the real negative sample, *False Negative (FN)* is predicted negative the real positive samples and *True Negative (TN)* is predicted negative the real negative sample.

## Results

This paper targets to develop a framework that can efficiently acquire the robust representation from the raw data for ransomware detection. In this study Citroni, CryptLocker, Cryptowall, Kollah, Kovter, Locker, and Matsnu families of ransomware were used for training and validation, and Pgpcoder, Reveton, TeslaCrypt and Trojan Ransomware were used for testing. Ransomware detection is tedious task due to varying underlying distribution and data scarcity. Hence, we offered a multi-phase framework, “CSPER,” for ransomware detection. The phases of proposed framework can be divided as: deep features extraction and ransomware detection. Ransomware detection phase is subdivided into: (1) base estimators training, (2) estimators selection and, (3) estimators aggregation strategy. The generalization aptitude is evaluated by weighing the proposed method on zero-day ransomware. Overall the performance of the proposed method is evaluated on error measures and recall. Evaluation with former related approaches is also accompanied and discussed.

### Effectiveness of different feature spaces

**Figure 8:**
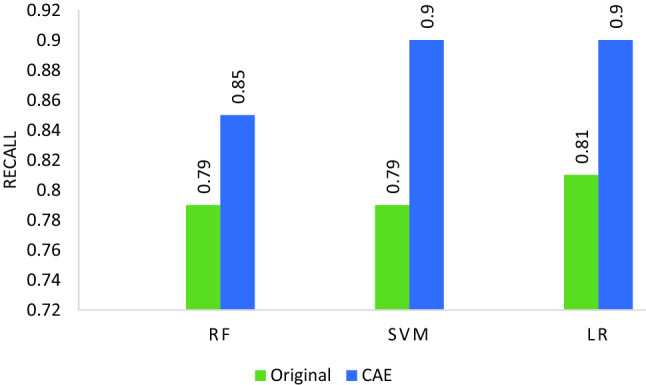
Comparison of original features vs. CAE reduced transformation on zero-day test data.

In this section, we will show the effectiveness of using CAE based feature spaces. Figure 8 shows the effectiveness of reduced representation achieved using CAE based transformation on three base learners SVM, RF, and LR. The feature size of the reduced representation shown is 100. It can be observed that all base estimators trained on reduced latent features are showing improved recall performance (CFH-RF (0.85), CFH-LR (0.90), CFH-SVM (0.90)) compared to the recall performance (RF (0.79), LR (0.81), SVM (0.79)) on original features. Consequently, the CAE can bring core discriminative representation by which all base estimators can perform better. It is due to the optimal selection of the penalty parameter of the CAE loss function that suppresses the variations of the ransomware variants.

**Figure 9:**
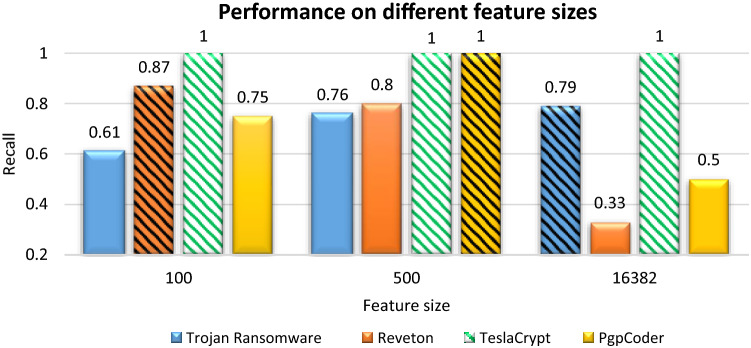
Comparison of different feature subspaces against zero-day test data.

The results for the selection of different feature sizes are visualized in Fig. 9. It shows the effect of zero-day attacks on two reduced set of features (100 and 500) and a complete set of original features (16,382) trained on RF. A feature reduction is carried out by training CAE by varying the number of hidden neurons and careful selection of penalty parameters in the validation phase.

The bars filled with patterns are showing the best performer feature size for a particular variant of ransomware. It can be observed that Reveton is showing an improved detection rate on the 500 feature set. Whereas, Pgpcoder detection is showing the better result on 100 feature set. However, Trojan Ransomware is better detected by a complete set of original features. Therefore it is proven that one single reduced feature set is not enough to detect all zero-day attacks.

Consequently, it shows that optimal feature selection, on the one hand, improves the results on validation and test data. While, on the other hand, it may lose the generalization ability, if the novel attack shares characteristic with the eliminated feature. Considering the unkown attack diversity, original feature set is also included to capture the whole behaviour. These selected feature spaces are used in the next phase for weighing the performance of the individual base estimators.

**Figure 10:**
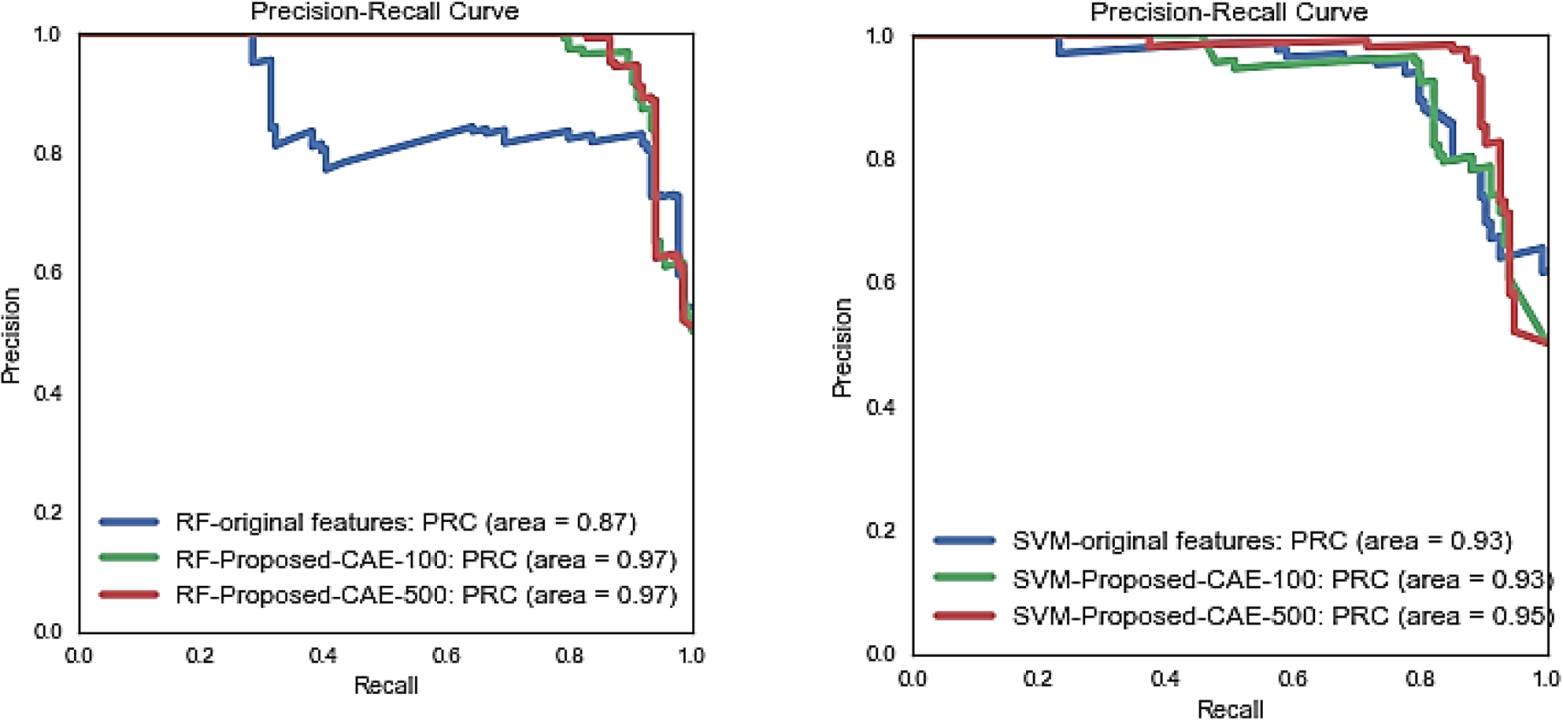
PR-Curve of different feature spaces.

The performance comparison of CAE based feature reduction of different sizes (CFH-RF(100) = 0.97, CFH-RF(500) = 0.97, CFH-SVM(100) = 0.93, and CFH-SVM(500) = 0.95) with original feature (RF(100) = 0.87, SVM(100) = 0.93) shown in Fig. 10 recommends that the proposed feature extraction method is worthy in detection of ransomware with significant AUC of PR curve for CAE based features. The prime objective is that the proposed method “CSPE-R” shows good generalization when applied to unknown feature representation and sizes trained on different decision surfaces.

### Effectiveness of using the proposed ensemble selection strategy

The proposed Pareto ensemble selection module shows a vital part in refining the overall results of CSPE-R ensemble method. To assess its effectiveness we compare the performance of the CSPE-R system using all base estimators vs. using selected base estimators (Results are shown in Table 3). Based on this experiment, it can be observed that CSPE-R has achieved the lowest false positive error (FP = 17) using Pareto estimator selection Strategy. This is due to the proposed Pareto-optimal constraints that efficiently exclude the weak base estimators. These constraints define the priority and desired compromise that we want to achieve for each error measure. Front 1 is representing the results of the selected estimators and front 2 is representing the results of the excluded estimators. Ensemble of front 2 estimators possess highest FP (40) and Net-err (41). Therefore, the proposed Pareto estimator selection module selects only the competent estimators.

**Table 3:** Comparison of the proposed ensemble method with and without using classifier selection strategy.

Methods	TN	FP	FN	TP	Net-err	Recall	Acc(%)
Proposed method with ensemble selection	116	17	1	133	18	0.99	93.0
Proposed method without ensemble selection	94	39	0	134	39	1.00	85.3
Front 1	116	17	1	133	18	0.99	93.0
Front 2	93	40	1	133	41	0.99	84.6

### Comparison with base estimators

This section evaluates the classification performance of the proposed ensemble classifier against baseline estimators, namely RF, SVM, and LR. An effective ensemble technique should ensure the accurate and diverse base learner. The proposed ensemble learning technique is used to detect the zero-day ransomware attacks in which different base-learners are used to construct diverse hypothetical spaces. The decision of a set of hypothetical spaces is more robust than a single hypothesis space. The proposed ensemble of “CSPE-R ” results in improved performance (as shown in Table 4) in terms of F1-score (0.93), recall (0.99), and discrimination ability (AUC: 0.93) as compared to the baseline estimators. Consequently, ensemble learning is more generalized than individual learners as an ensemble can reach multiple sub-optimal spaces.

**Table 4:** Comparison of the proposed ensemble with individual base learners concerning Accuracy, Recall, and F1-score on zero-day test data.

Methods	Accuracy	Recall	F1
Proposed CFH-SVM(100 )	0.90	**0.90**	0.90
Proposed CFH-RF( 100)	**0.92**	0.84	0.91
Proposed CFH-LR(100)	0.90	0.89	0.90
Proposed CFH-SVM(500)	**0.92**	0.87	**0.92**
Proposed CFH-RF(500)	0.89	0.79	0.88
Proposed CFH-LR(500)	0.89	0.87	0.89
SVM	0.88	0.79	0.87
RF	0.80	0.76	0.79
LR	0.90	0.81	0.89
Proposed CSPE-R Ensemble	**0.93**	**0.99**	**0.93**

The black bold values show the top-1 results.

### Comparison with the existing techniques

To assess the performance of the CSPE-R detection system contrary to the existing techniques, we consider different methods from the current studies. The Table 5 compares our proposed methods and the other methods, namely DNAact-Ran^40^, OC(SVM)^16^, EldeRan^35^, and SA-CNN. DNAact-Ran practices Digital DNA Sequencing Engine for data representation and active learning for ransomware detection. *One Class SVM (OC (SVM))* is an anomaly based method trained on benign samples to detect novel ransomware. EldeRan detects ransomware by selecting dynamic features such as registry key operations, APIs call, file system activities using mutual information criteria and classify each sample using LR classifier. For a fair comparison, we selected the EldeRan^35^ and DNAact-Ran^40^ techniques that are using the same dataset. The results show that the proposed technique is superior in detecting ransomware as compared to the existing state of the art methods. This superiority is due to the cost-sensitive base estimators, the proposed cost-sensitive ensemble selection strategy and the cost-sensitive combination rule used in the proposed technique.

**Table 5:** Comparison with current techniques.

Athors	Method	Recall	Test data
Our proposed	CSPE-R	0.99	Unknown attacks
Khan et al.^40^	DNAact-Ran OC(SVM)	0.82	Known attacks
Al-rimy et al.^16^	Anomaly	0.89	Unknown attacks
Sgandurra et al.^35^	EldeRAN	**0.93**	UnKnown attacks
Zhang et al.^41^	SA-CNN	0.87	Known attacks

Significant values are in [bold].

### Comparison with state of the art methods

**Figure 11:**
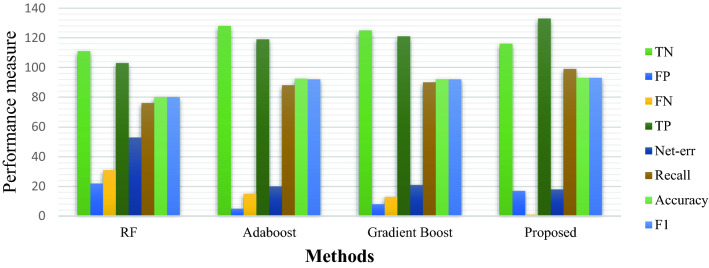
Comparison between state of the art ensemble methods.

In this section, we compare the proposed heterogeneous CSPE-R ensemble method with the state of the art homogeneous ensemble learning methods. GradientTreeBoost^42^ is an ensemble of weak learners that are added to minimize the gradient loss function to boost the generalization performance. Adaboost is an Adaptive boosting technique that is an ensemble of decision stumps that assigns different weights to base trees unlike RF). Then, in view of the benefits of Adaboost, we also compared the Adaboost and RF. Figure 11 shows that our CSPE-R method has higher recall, accuracy, F1, TP value together with lower FN.

### Statistical test

To assess the significance of the proposed CSPE-R ensemble method over its base estimators paired t-test is performed. The outcomes of the proposed technique for ten independent runs of recall measure are compared with each base estimator. The null hypothesis indicated no difference between the results of base estimators and the proposed CSPE-R ensemble. The results are tabulated in Table 6 for all the estimators. It can be observed that for all of the base estimators, p-value is less than the critical value 0.01, which indicates rejection of the null hypothesis. This in turn, shows improvement in diversity and thus the generalization capability of proposed CSPE-R ensemble techniques over its base estimators.

**Table 6:** Paired t-test comparison with base estimator on test data.

Estimator’s p-values	RF	SVM	LR	CFH-RF(100)	CFH-SVM(100)	CFH-LR(100)	CFH-RF(500)	CFH-SVM(500)	CFH-LR(500)
CSPE-R Ensemble	1.97E−15	9.29E−17	2.30E−16	1.62E−11	8.07E−14	3.37e−14	2.32E−16	8.07E−14	3.37E−14
Hypothesis test	1	1	1	1	1	1	1	1	1

## Discussion

In this work, we have developed a deep features based multi-phase ransomware detection framework “CSPER”. The uniform robust features representation can facilitate the cyber-security experts to generate the dynamic features of any unknown attacks in an unsupervised manner.

In this study, we exploited the contractive autoencoder to suppress the little changes around different families of ransomware and goodware to find core embeddings. The proposed contractive pressure based deep feature extraction overcomes the limitations of existing feature reduction and extraction techniques used by different researchers, which resulted in high false-positive and ignored true positives. Principal Component Analysis PCA, is unable to find principal component if there exist no relation between features. Similarly, CorrAUC^43,44^ is a correlation based wrapper feature selection method developed to detect the malicious traffic. Simple autoencoder based feature reduction techniques presented in literature are highly sensitive to noisy data. Whereas, De-noising autoencoders can better deal with noisy data by producing the noisy input to achieve identity function, during reconstruction tries to recover original input. This may not work as the noise distribution changes. Whereas, CAE’s can explicitly encourage robustness, which is more vibrant as it penalizes large derivatives at training data. This characteristic makes it superior to other choices.

In this research deep Contractive Autoencoder is exploited to extract multiple core embeddings which facilitate more information transfer from known class of ransomware to unknown class of ransomware.

To address minority class and class imbalance problem this research work showed that with a combination cost-sensitive base classifiers and model-tuning using cost matrix obtained while validation process can effectively reduce the effects of class imbalance issue.

Furthermore, in order to achieve a high recall and low FN our proposed scheme exploits Parito-optimality based non-dominant sort to select the suitable learners for final prediction. A non-dominated base learner is a base learner that is not inferior to any other base learner and is superior to as a minimum of one base learner, which means that there is no base learner to dominate it. That is why it has the ability to select only feasible base classifiers for final decision aggregation. Proposed Pareto-optimality based selection strategy is guided by FN and Net-Error. This helps in achieving a cost sensitive compromise among FP and FN errors. As low FN is our top priority, but it may be the case that a model with very low FN may not be learning. Therefore, we put constraint to select only those models with low FN that are also having simultaneous decrease in its net error. Through different experiments, we showed that post-processing tools like aggregation operator of ensemble schemes have a vital role in selecting the correct prediction. Also, to achieve a better tradeoff between FP and FN a decision must be made according to the domain under consideration.

To evaluate the efficacy of the proposed solution in comparison to the existing techniques, quantitative evaluation of the proposed techniques has been performed in comparison to different state of art methods, and conventional learning methods. Both FN and recall are considered as important parameters to show the efficacy of the proposed techniques, additionally, accuracy, F1 score, FP, TP and TN were adopted as evaluation parameters.

## Conclusion

Ransomware is one of the highly threatening malwares and is difficult to detect because of continuous increase in its new variants. Thus, IDS trained for specific ransomware events often results in suboptimal performance for new adaptations of ransomware (zero-day) attacks. In this work, a new ensemble learning strategy, “CSPE-R” is developed to address the challenge of heterogeneous behaviour of ransomware attacks by aggregating the discrimination ability of multiple-experts and considering varied events. In this regard, we explored diverse semantic spaces, exploited cost-sensitive optimization, and developed a new Pareto-optimality based base-estimator selection strategy to improve the zero-day ransomware detection performance. The proposed ensemble selection strategy effectively improves the generalization performance against zero-day ransomware attack by gaining improvement in recall (9%) and F1-score (1%), when compared to the best performing base estimator and other ensemble learning strategies. The proposed idea of learning dissimilarities between the different families of the attacks and subsequent development of a cost sensitive based ensemble for the detection of zero-day ransomware attacks has the potential to be applied for other types of zero-day attacks as well.

The proposed framework considers host based features, however in future, network traffic verification can also be investigated as a features. Additionally, the current study focusses on eleven families of ransomware, extended version may comprise of training the proposed framework with additional ransomware variants. Dynamic analysis is a time consuming activity. In future, this feature extraction time can be reduced by investigating only pre-encryption based features.

## Data Availability

The dataset used in this research work has been downloaded from the website https://rissgroup.org/ransomware-dataset/.Detailed, detailed description of the data set is provided in dataset section.

## References

[1] Al-rimy BAS, Maarof MA, Shaid SZM (2018). Ransomware threat success factors, taxonomy, and countermeasures: A survey and research directions. Comput. Secur..

[2] Bridges L (2008). The changing face of malware. Netw. Secur..

[3] Bhardwaj A, Avasthi V, Sastry H, Subrahmanyam GVB (2016). Ransomware digital extortion: A rising new age threat. Indian J. Sci. Technol..

[4] FBI Anouncements, P. S. In *FBI.Criminals continue to defraud and extort funds from victims using CryptoWall Ransomware scheme* (2015).

[5] Kaspersky. In *KSN Report:Ransomware in 2014–2016 Kasperkey Lab* (2016).

[6] Kim G, Lee S, Kim S (2014). A novel hybrid intrusion detection method integrating anomaly detection with misuse detection. Expert Syst. Appl..

[7] Or-Meir O, Nissim N, Elovici Y, Rokach L (2019). Dynamic malware analysis in the modern era—a state of the art survey. ACM Comput. Surv..

[8] Ahmadian, M. M., Shahriari, H. R. & Ghaffarian, S. M. Connection-monitor & connection-breaker: A novel approach for prevention and detection of high survivable ransomwares. In *12th International ISC Conference on Information Security and Cryptology, ISCISC 2015*. 10.1109/ISCISC.2015.7387902 (2016).

[9] Suresh, S., Mohan, M., Thyagarajan, C. & Kedar, R. Detection of ransomware in emails through anomaly based detection. In *Lecture Notes on Data Engineering and Communications Technologies* (2020).

[10] Thabtah F, Hammoud S, Kamalov F, Gonsalves A (2020). Data imbalance in classification: Experimental evaluation. Inf. Sci. (NY).

[11] Khan A, Sohail A, Zahoora U, Qureshi AS (2019). A survey of the recent architectures of deep convolutional neural networks. Artif. Intell. Rev..

[12] Xu D, Tian Z, Lai R, Kong X, Tan Z, Shi W (2020). Deep learning based emotion analysis of microblog texts. Inf. Fusion.

[13] Kaur R, Singh M (2014). A survey on zero-day polymorphic worm detection techniques. IEEE Commun. Surv. Tutorials.

[14] Fagioli A (2019). Zero-day recovery: The key to mitigating the ransomware threat. Comput. Fraud Secur..

[15] Al-rimy, B. A. S., Maarof, M. A. & Shaid, S. Z. M. In *A 0-day Aware Crypto-Ransomware Early Behavioral Detection Framework* (2018).

[16] Al-rimy BAS, Maarof MA, Prasetyo YA, Mohd-Shaid SZ, Mohd-Ariffin AF (2018). Zero-day aware decision fusion-based model for crypto-ransomware early detection. Int. J. Integr. Eng..

[17] Zhu J, Jang-Jaccard J, Singh A, Welch I, AI-Sahaf H, Camtepe S (2022). A few-shot meta-learning based siamese neural network using entropy features for ransomware classification. Comput. Secur..

[18] Wang P, Tang Z, Wang J (2021). A novel few-shot malware classification approach for unknown family recognition with multi-prototype modeling. Comput. Secur..

[19] Masdari M, Khezri H (2020). A survey and taxonomy of the fuzzy signature-based Intrusion Detection Systems. Appl. Soft Comput. J..

[20] Sreelaja NK (2021). Ant colony optimization based light weight binary search for efficient signature matching to filter ransomware. Appl. Soft Comput..

[21] Schultz MG, Eskin E, Zadok E, Stolfo SJ (2001). Data mining methods for detection of new malicious executables. Proc. IEEE Comput. Soc. Symp. Res. Secur. Priv..

[22] Shabtai A, Moskovitch R, Elovici Y, Glezer C (2009). Detection of malicious code by applying machine learning classifiers on static features: A state-of-the-art survey. Inf. Secur. Tech. Rep..

[23] Young AL (2006). Cryptoviral extortion using Microsoft’s Crypto API. Int. J. Inf. Secur..

[24] Andronio N, Zanero S, Maggi F (2015). HELDROID: Dissecting and detecting mobile ransomware. Lecture Notes Comput. Sci..

[25] Taheri R, Ghahramani M, Javidan R, Shojafar M, Pooranian Z, Conti M (2020). Similarity-based Android malware detection using Hamming distance of static binary features. Futur. Gener. Comput. Syst..

[26] Das S, Xiao H, Liu Y, Zhang W (2016). Online malware defense using attack behavior model. Proc. IEEE Int. Symp. Circ. Syst..

[27] Tajoddin A, Abadi M (2019). RAMD: Registry-based anomaly malware detection using one-class ensemble classifiers. Appl. Intell..

[28] Stolfo SJ (2005). A comparative evaluation of two algorithms for Windows Registry Anomaly Detection. J. Comput. Secur..

[29] Luo C, Tan Z, Min G, Gan J, Shi W, Tian Z (2021). A novel web attack detection system for internet of things via ensemble classification. IEEE Trans. Ind. Inform..

[30] Ding Y, Chen S, Xu J (2016). Application of deep belief networks for opcode based malware detection. Proc. Int. Joint Conf. Neural Netw..

[31] Tian Z, Luo C, Qiu J, Du X, Guizani M (2020). A distributed deep learning system for web attack detection on edge devices. IEEE Trans. Ind. Inform..

[32] Sohail A, Khan A, Wahab N, Zameer A, Khan S (2021). OPEN A multi-phase deep CNN based mitosis detection framework for breast cancer histopathological images. Sci. Rep..

[33] Zhou Z, Kearnes S, Li L, Zare RN, Riley P (2019). Optimization of molecules via deep reinforcement learning. Sci. Rep..

[34] Talwar D, Mongia A, Sengupta D, Majumdar A (2018). AutoImpute: Autoencoder based imputation of single-cell RNA-seq data. Sci. Rep..

[35] Sgandurra, D., Muñoz-González, L., Mohsen, R. & Lupu, E. C. In *Automated Dynamic Analysis of Ransomware: Benefits, Limitations and use for Detection* (2016).

[36] Boehmke, B., Greenwell, B., Boehmke, B. & Greenwell, B. Autoencoders,” in *Hands-On Machine Learning with R*, 2020.

[37] Rifai, S. *et al*. Higher order contractive auto-encoder. In *Lecture Notes in Computer Science (including subseries Lecture Notes in Artificial Intelligence and Lecture Notes in Bioinformatics)*. 10.1007/978-3-642-23783-6_41 (2011).

[38] Giacinto G, Perdisci R, Del Rio M, Roli F (2008). Intrusion detection in computer networks by a modular ensemble of one-class classifiers. Inf. Fusion.

[39] Muhammad MGS, Tian Z, Sun Y, Du X (2020). Selection of effective machine learning algorithm and Bot-IoT attacks traffic identification for internet of things in smart city. Futur. Gener. Comput. Syst..

[40] Khan F, Ncube C, Ramasamy LK, Kadry S, Nam Y (2020). A digital DNA sequencing engine for ransomware detection using machine learning. IEEE Access.

[41] Zhang B, Xiao W, Xiao X, Sangaiah AK, Zhang W, Zhang J (2020). Ransomware classification using patch-based CNN and self-attention network on embedded N-grams of opcodes. Futur. Gener. Comput. Syst..

[42] Friedman BJH (2001). Greedy function approximation: A gradient boosting machine. Ann. Stat..

[43] Shafiq M, Tian Z, Bashir AK, Du X, Guizani M (2021). CorrAUC: A malicious Bot-IoT traffic detection method in IoT network using machine-learning techniques. IEEE Internet Things J..

[44] Shafiq M, Tian Z, Bashir AK, Du X, Guizani M (2020). IoT malicious traffic identification using wrapper-based feature selection mechanisms. Comput. Secur..

